# Validating Causal Diagrams of Human Health Risks for Spaceflight: An Example Using Bone Data from Rodents

**DOI:** 10.3390/biomedicines10092187

**Published:** 2022-09-05

**Authors:** Robert J. Reynolds, Ryan T. Scott, Russell T. Turner, Urszula T. Iwaniec, Mary L. Bouxsein, Lauren M. Sanders, Erik L. Antonsen

**Affiliations:** 1 KBR Wyle Services, LLC, NASA Johnson Space Center, Houston, TX 77058, USA; 2 KBR, Space Biosciences Division, NASA Ames Research Center, Moffett Field, CA 94043, USA; 3 Skeletal Biology Laboratory, Oregon State University, Corvallis, OR 97331, USA; 4 Center for Advanced Orthopaedic Studies, Beth Israel Deaconess Medical Center, Boston, MA 02215, USA; 5 Department of Orthopedic Surgery, Harvard Medical School, Boston, MA 02115, USA; 6 Blue Marble Space Institute of Science, Space Biosciences Division, NASA Ames Research Center, Moffett Field, CA 94043, USA; 7 Department of Emergency Medicine, Center for Space Medicine, Baylor College of Medicine, Houston, TX 77030, USA

**Keywords:** spaceflight, microgravity, causal diagrams, directed acyclic graphs, bone fracture, translational science

## Abstract

As part of the risk management plan for human system risks at the US National Aeronautics and Space Administration (NASA), the NASA Human Systems Risk Board uses causal diagrams (in the form of directed, acyclic graphs, or DAGs) to communicate the complex web of events that leads from exposure to the spaceflight environment to performance and health outcomes. However, the use of DAGs in this way is relatively new at NASA, and thus far, no method has been articulated for testing their veracity using empirical data. In this paper, we demonstrate a set of procedures for doing so, using (a) a DAG related to the risk of bone fracture after exposure to spaceflight; and (b) four datasets originally generated to investigate this phenomenon in rodents. Tests of expected marginal correlation and conditional independencies derived from the DAG indicate that the rodent data largely agree with the structure of the diagram. Incongruencies between tests and the expected relationships in one of the datasets are likely explained by inadequate representation of a key DAG variable in the dataset. Future directions include greater tie-in with human data sources, including multiomics data, which may allow for more robust characterization and measurement of DAG variables.

## 1. Introduction

The risks to human health and performance in spaceflight are myriad and complex [1,2,3,4,5]. The work involved in characterizing and mitigating those risks draws from highly disparate groups of expertise, including life scientists, physicians, engineers, and program managers. The de facto approach to both the research and management of spaceflight human health risks has been to consider the major risks as isolated problems, with little to no relation to the others [4]. This allowed deep exploration by specific sets of experts in the characterization phases but introduced challenges into the translation to clinical and operational domains where both logic and experience tell us that these risks are interconnected, with risk systems sharing common causes and contributing to common outcomes [3,4]. Effective reduction in the risks humans face in spaceflight requires translation of the knowledge gained in areas such as space biology to improve mitigations and countermeasures at the human level. These translational challenges are experienced in different forms across many fields [6]. The attempt to develop countermeasures to reduce the likelihood of one potential problem may lead to the unintended increase in the probability of others. To approach and manage this complexity, the Human Systems Risk Board (HSRB) at the National Aeronautics and Space Administration (NASA) embarked on a pilot project creating a series of causal diagrams that explicitly graph the pathways between known and suspected contributing factors at the highest level, and the sequence of events between them. These pathways all start with exposure to spaceflight environmental hazards, and end with mission level outcomes of interest to the astronauts and the agency. One narrative causal diagram was created for each of 29 individual spaceflight risks [5,7]. Examples of HSRB risks include those organized around hazardous exposures (e.g., Toxic Exposure Risk, Celestial Dust Risk, Radiation Risk), those centered on medical outcomes (e.g., Bone Fracture Risk, Venous Thromboembolism Concern, Spaceflight-Associated Neuro–Ocular Syndrome), and those concerned with in-flight deconditioning (e.g., Muscle Risk, Aerobic Risk). Insight from these areas helps inform how to set standards and adjust design to accommodate the human in the spaceflight vehicles, habitats, and spacesuits. Thus, the interconnected relationship among risks extends to Human Systems Integration Architectures with the attendant increase in system complexity.

The causal diagrams used in conjunction with the HSRB risks are created and maintained as Directed Acyclic Graphs (DAGs), a type of network diagram with properties well-suited to causal diagramming [8,9]. The resultant DAGs facilitate better communication of complex risks, and the structure of the fully integrated risk network allows for insights generated through social network type analysis and leaves open the possibility of evolving the combined risk network to a Bayesian Network.

The creation of causal diagrams via subject matter expert opinion does not guarantee accuracy of the causal explanations they contain. In fact, one goal of creating and configuration managing these diagrams by the HSRB is to invite evidence-based recommendations for evolving the diagrams. Without the addition of empirical data to back the causal explanations on a diagram, it is only speculation—however well-informed it may purport to be. These DAGs, and the validation of DAGs (with data underpinning their structural relationship), will ultimately be used by mission planners and NASA leadership to create systemic quantifiable biomedical health risk probabilities for upcoming deep space missions (i.e., cis-Lunar, Mars transit, Mars surface missions). Once empirical data populate the many risks via DAGs, and those DAGs are validated, approaches are enabled for mission planners to receive concrete individualized probability values of adverse health risks on deep space missions. It must be made clear at this point that the currently available quantified probabilities for mission planners are limited to the Integrated Medical Model and the NASA Space Cancer Radiation model, which together address only 2 of the 29 HSRB risks (the Medical Conditions Risk and the Radiation Carcinogenesis Risk) [10,11,12]. Mission planners currently proceed with the best information that is available, but there is a strong need to develop innovative approaches for quantifying systemic risk in an evidence-based manner.

As causal diagrams are relatively new to the biomedical field at NASA, there has not yet been the exposition of a method and procedure for using data to validate them. An objective and repeatable procedure is needed so that researchers both inside and outside NASA can use data in a standardized, meaningful fashion for validating, and as necessary, proposing amendments to the causal explanations encapsulated in the 29 risk diagrams. An ideal method is one that can be generalized to utilize data from various organisms (humans, animals, or cells), and from either spaceflight or terrestrial analogue exposures. These data from various levels of evidence (astronaut clinical, human research, animal, cellular) are scored at varying levels of strong, moderate, weak, or speculative in the greater DAG data schema [5]. Here we expand upon the framework put forth by Ankan et al. [8] and propose that this method be used for translating space or analog data into evidence either in support of or in refutation of a causal diagram. We illustrate the method using data from a limited portion of the HSRB Bone Fracture Risk DAG, with data from four studies of rodent bone turnover from either spaceflight or a partial weightbearing exposure analog. This paper serves as an exploration of methods to validate DAGs as well as a pathfinder for approaches to translating valuable human and animal research data into a domain that is intended to inform risk reduction in human spaceflight.

## 2. Materials and Methods

Causal diagrams attempt to visually represent the cause-and-effect relationships between multiple factors or events. DAGs are a form of network graph that have requirements and properties making them useful for causal representation [8,9]. DAGs are inherently visual (graph), have one-way links (directed), and contain no loops (acyclic). These latter two requirements of DAGs are important as they are what establishes and enforces the temporal relationship between variables: causes must precede effects. 

The objects on DAGs are often called “nodes” or “vertices” and they represent random variables. Causal relationships between variables are represented as directed (one-way) arrows, originating with causes and terminating in effects. These arrows are referred to as “links” or “edges”.

In this framework, one factor is a “cause” of another if two conditions are met: the cause temporally precedes the effect, and the probability distribution of the effect varies over the values of the cause. Note that this is equivalent to saying that the variables in the cause–effect relationship are not statistically independent from one another. This also means that we can consider each node to have a conditional probability distribution inside of it, which depends on the values assumed by all its causes. It is this probabilistic relationship between causes and effects which we will exploit in the validation of causal diagrams.

The causal diagram we present as our example here is a slightly modified subgraph extracted from a larger causal diagram developed by scientists at NASA to explain the risk of skeletal fragility in response to changes in skeletal loading under conditions of altered gravity [7]. This example is the first attempt to populate and validate with empirical data any section of the 29 HSRB-created space health risk DAGs.

Data were obtained from the NASA Ames Life Science Data Archive (ALSDA) [13]. In 2021, ALSDA integrated with NASA GeneLab [14], and began scientifically curating datasets in-line with NASA GeneLab standards (a multiomics-centric repository), thus enabling scientific spaceflight and space-relevant data to be minable concurrently (both multiomics and phenomic–physiological–behavioral). In addition, this integrative ALSDA transformation ensured data quality and scientific confidence in datasets accessed by researchers and the public for reuse. This also included ensuring ALSDA datasets were maximally open access [15], FAIR (findable, accessible, interoperable, reusable) [16], and data files were curated to be machine-readable (e.g., “tidy” data format) [17], transparent, reproducible, and maximally “AI-ready” [13,18,19]). 

This study’s method for DAG validation for the Bone HSRB-produced risk involves four datasets from rodents [20,21,22,23] produced through the bioassays of: (1) micro-computed tomography (μCT), (2) dual-energy X-ray absorptiometry (DXA), (3) histomorphometry, or (4) biomechanical strength testing [24,25,26,27]. Values derived from the studies pertained to one or more of: bone formation (mineral apposition rate, ceased bone formation, mineralized surface/bone surface, and bone formation rate/bone surface); bone resorption (label length and osteoclast perimeter); bone mass (bone volume/tissue volume, bone mineral content, and bone mineral density); trabecular microarchitecture (trabecular thickness, number, and separation/spacing); or bone strength (maximum load/fail load). Table 1 provides details of these studies, including the number of animals, study conditions, and measurements made. The study by Dubeé et al. contained data from μCT only; the 2015 Keune et al. study provided data from both μCT and DXA; the 2016 Keune et al. study offered measurements from histomorphometry; and Ko et al. had data obtained by μCT, histomorphometry, and biomechanical strength testing data. 

Three of the included studies were of rats [21,22,23] and one was of mice [20]. There was one ground-based study which used partial body weight unloading as the exposure, while the other three studies sent animals to space. The design of the spaceflight studies was generally one exposure group with one or more terrestrial control groups. Total sample sizes of the studies were approximately 24 animals, save for the ground analog study of Ko et al., where rats were randomized to one of four partial weightbearing levels and one of three exposure durations, for a total of 12 exposure groups. Each exposure condition had 12 rats assigned to it, for a total of 144 animals. However, due to missing data, the working dataset had complete observations on only 140 rats. 

To avoid adding variables to the bone DAG used here, to make the data comparable to the spaceflight studies, and to ensure any bone effects due to unloading were clearly observable in the data, we included only the 0% (control group), 60%, and 80% unloaded exposure groups, and only those animals that were exposed for 28 days. This resulted in a dataset of 33 observations from the Ko et al. study. Of those 33, 14 had measures of bone formation and bone resorption, making the effective sample size for the tests of conditional independencies only 14. Thus, the tests of marginal correlation had a sample size of either 33 or 14, depending on whether or not they were testing a relationship that included bone formation or resorption. 

The original datasets contained variables that were related to—but not necessarily synonymous with—the random variables on the bone DAG. In most cases, the data contained more than one variable related to single concepts on the causal diagram. As a method of using all available information from multiple measures in a compact way, we created composite variables using principal component analysis (PCA). The details of this procedure and its results can be found in the Supplementary Materials to this article.

The statistical analyses in this study consisted of building composite measures of the random variables on the bone causal diagram and then using those measures to test relationships implied by the diagram. The testing procedure for these relationships generally follows the approach set out by Ankan et al. [8].

Through structural analysis of the bone risk DAG, we extracted the set of expected marginal correlations and conditional independencies implied by the structure of the DAG [9,28]. These serve as testable hypotheses for the accuracy of the network structure. Marginal dependencies are pairs of variables that should have a non-zero bivariate correlation (i.e., when not controlling for other factors). Conditional independencies are pairs of variables that should have zero correlation when controlling for one or more variables as specified by analysis of the DAG structure. To define the variable relationships implied by the bone risk DAG, we used the dagitty web interface (available at dagitty.net) and the package “dagitty” in the R statistical computing software, version 2021.09.2, Vienna, Austria [8,29]. To compute marginal correlations between pairs of variables we used the “ci_cor” function in the “confintr” package [30]. This function computes the correlation as well as the 95% confidence interval for the correlation. To guard against violations of linearity between pairs of variables, we specified the function to use the Spearman correlation statistic. Confidence intervals for the Spearman correlation coefficient were generated with bias-corrected accelerated bootstrapping, the default for the package. This method adjusts the endpoints of the bootstrapped confidence interval to compensate for skew among the bootstrapped samples, leading to more robust estimates.

The “dagitty” package in R offers the function “localTests”, which takes both a DAG (in the form of dagitty code) and the correlation matrix of variables that correspond to the DAG’s variables as its input. The function then returns partial correlations for each conditional independency in the DAG and their 95% confidence intervals. We used this function to compute the set of correlations corresponding to the conditional independencies on the DAG.

Whereas marginal correlations are pairs of variables expected to have non-zero correlation, conditional independencies are relationships where the partial correlation is expected to be 0 after controlling for specific intermediate variables along causal pathways (and if the DAG is correct). We considered a correlation to be non-zero if it passed both of the following criteria: (a) the correlation is the direction (positive or negative) consistent with the posited causal relationships; and (b) the absolute value of the correlation is greater than or equal to 0.15. In other words, a marginal correlation is validated as non-zero if it is directionally correct and is of sufficient magnitude to be distinguishable from 0. Partial correlations generated to test conditional independencies are validated if the absolute value of the partial correlation coefficient is less than 0.15 (i.e., is indistinguishable from 0). We chose a threshold of magnitude for non-zero correlation because it is possible to have values that are close to 0 with tight confidence intervals that do not include 0. In such instances the correlation is effectively zero, and the magnitude threshold allows us to interpret it as such. We chose the value of 0.15 as a minimal heuristic standard. As such, this value can be considered arbitrary, and other thresholds can and should be considered depending on the context.

Another possible validation criterion could be that the 95% confidence interval surrounding the correlation does not include 0. In our view, directionality and magnitude of correlation are far more important criteria in biological systems, and thus, together are sufficient to consider a variable pair to be correlated. Perhaps more importantly, Ggiven the potential lack of statistical power in spaceflight research, and recent criticisms of statistical hypothesis testing in spaceflight research [31,32], we believe that a lack of statistical precision alone should not be enough to disqualify a marginal correlation from being validated. As such, we do not consider the confidence interval to be a necessary ingredient for validating DAG structure, but report it here for comparison and discussion.

## 3. Results

### 3.1. Causal Diagram

**Figure 1:**
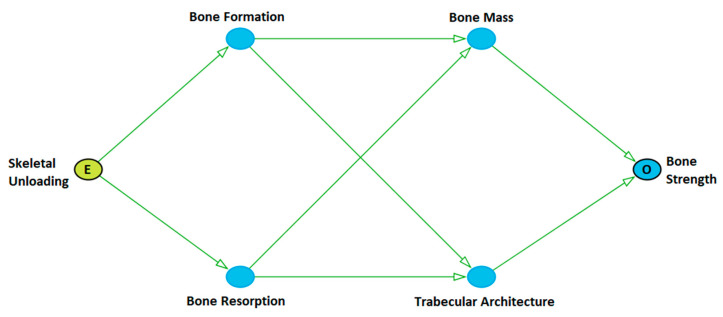
Causal diagram (in DAG form) showing the causal pathways between skeletal unloading and bone strength.

A modified portion of the bone risk DAG (i.e., a “subgraph”) is shown as Figure 1. In this causal diagram, skeletal unloading is the “exposure” as denoted by the green node color and the label “E”. As such, it is the ultimate cause of all downstream nodes through its direct causal influence on bone formation and bone resorption. These two processes are both causes of bone mass and trabecular architecture, which in turn are both causes of the outcome, bone strength, here marked with an “O”. The arrows between all variables are colored green, which denotes that all paths on this diagram are on causal paths between skeletal unloading and bone strength.

### 3.2. Variable Relationships

Analysis of the DAG in Figure 1 revealed the marginal correlations and conditional independencies implied by the diagram. Because of the general linear flow of the causation in the DAG, all variables on the DAG should have non-zero marginal correlation. Table 2 lists the pairs of variables which should be marginally correlated, and the expected direction (positive or negative) between them. In general, skeletal unloading should result in less bone formation (negative correlation) and more bone resorption (positive correlation). These in turn should lead to lower bone mass and a sparser trabecular microarchitecture. Lower mass and a sparser trabecular microarchitecture should result in lower bone strength.

Table 3 displays the conditional independencies implied by Figure 1. Careful study of the statements in Table 3 reveals that variables are conditionally independent whenever all paths between them are “blocked”. For example, bone strength is independent of exposure when we control for bone formation and bone resorption, or when we control for bone mass and trabecular microarchitecture. Similarly, exposure is independent of both bone mass and trabecular microarchitecture when we control for bone formation and bone resorption. 

Two variables on a DAG are also independent whenever we have accounted for their common causes. This is demonstrated when bone formation is independent of bone resorption after controlling for skeletal unloading, and with bone mass being independent of trabecular microarchitecture after controlling for bone formation and bone resorption.

### 3.3. Tests of Marginal Correlation

**Figure 2:**
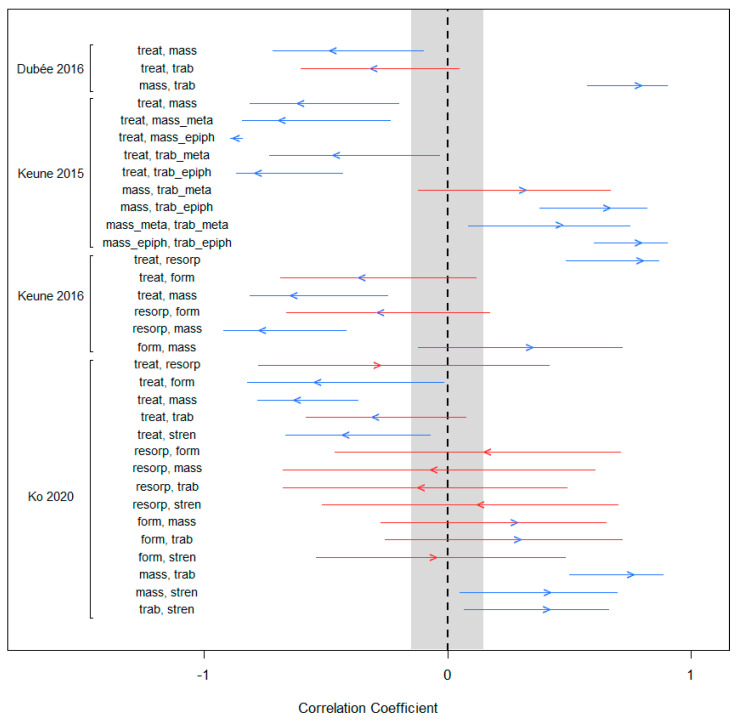
Correlations and 95% confidence intervals for testing marginal associations. The grey band represents the no-effect zone, or the zone in which a point estimate is considered indistinguishable from zero. Arrows point in the direction of expected correlation, above or below zero; blue arrows are those of sufficient magnitude to be considered non-zero (outside of the grey band), while red arrows are of insufficient magnitude to be considered non-zero (inside the grey band). Blue interval bands exclude 0 while red interval bands include 0.

The results of the tests of marginal correlation are displayed in Figure 2. Point values are correlation coefficients between the pairs of variables (marked with arrowheads), and the segments are their 95% confidence intervals. Blue arrowhead markers represent correlations whose absolute values are greater than or equal to 0.15—the required minimum value to be considered a non-zero correlation—and are in the expected direction of association (i.e., positive or negative). Blue confidence bands are those that do not intersect 0 (the dotted, vertical line in Figure 2). Those with red arrowhead markers have absolute values less than or equal to 0.15 and/or are not in the expected direction; those with red confidence bands are those that cross 0.

The bone DAG implied 15 marginal correlations among its six variables. Although the datasets used here enabled us to conduct 38 tests in all, not all studies offered tests of all relationships. Of note is the study by Ko et al., which offered the only measurement of bone strength, and thus the only set of tests of this variable against the others. 

Examination of Figure 2 reveals that all correlations from the Dubeé data were in the expected direction, and had sufficient magnitude of effect size to be considered non-zero correlations. The correlation between exposure and trabecular microarchitecture was the only correlation lacking statistical precision, with its confidence interval inclusive of zero.

The 2015 Keune data enabled us to test three relationships with nine marginal correlations. Once again, all of these correlations were in the correct direction and of sufficient effect size to be considered non-zero correlations. Here too, a single correlation lacked statistical precision.

The Keune 2016 data afforded six correlations. All six of the point estimates were in the expected direction and of sufficient magnitude to be considered non-zero. Three of these had confidence intervals that crossed zero. 

The Ko data provided the opportunity to compute 15 correlations. Among these, four fell within the grey zone in Figure 2, making them effectively zero. Two correlations had point values outside the grey zone but were in the wrong direction. The remaining nine correlations were directionally correct and large enough to be considered non-zero. Three of them had confidence intervals inclusive of 0.

### 3.4. Tests of Conditional Independencies

**Figure 3:**
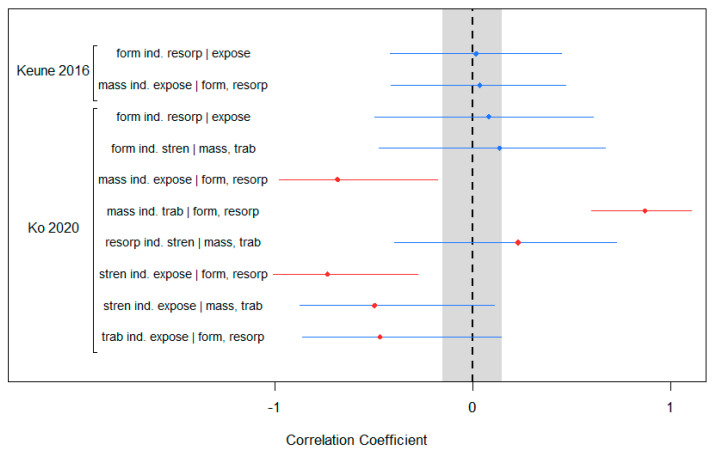
Partial correlations and 95% confidence bands for testing conditional independencies. The grey band represents the no-effect zone, or the zone in which a point estimate is considered indistinguishable from zero. Blue points are those that lie inside the grey band and thus are considered to have zero correlation. Red points are those that lie outside the grey band and thus are considered to have non-zero correlation. Blue interval bands include 0 and red bands exclude 0.

The bone DAG of Figure 1 offered eight conditional independencies, but only the Keune 2016 and the Ko datasets contained variables that would allow for testing them. The Ko data were able to test all eight conditional independencies, while the Keune 2016 data were able to test two of them. Figure 3 displays the results of these tests.

The Keune 2016 data offered tests of the independence of bone formation from bone resorption given exposure, and the independence of bone mass from exposure given bone formation and bone resorption. Both correlations were within the −0.15 to 0.15 limits effectively validating these two conditional independencies in those data.

In the Ko data, only two out of eight conditional independencies were validated. Among the six non-zero correlations, all of them involved either bone resorption, bone formation, or both. Four of the failed tests were conditional on both bone formation and bone resorption, which was the entire set of relationships that were conditional on this pair. One of the other two non-zero results also involved bone formation but not resorption, and the other involved bone resorption but not formation. The relationships that were confirmed included the conditional independence of the exposure and bone strength when controlling for bone mass and trabecular microarchitecture, and the independence of bone formation and resorption when controlling for exposure.

## 4. Discussion

### 4.1. Interpretation of Findings

In this example DAG validation, we used data from four studies of the effects of skeletal unloading from rodents to test the causal explanation of how unloading may lead to diminished bone strength. Our results were mixed in that the marginal correlations provided some evidence for the involvement of all the included variables in the story of skeletal unloading and bone strength, but six of the eight conditional independencies were not validated by the data. At first blush, this might lead us to reject at least some of the structure of the DAG presented as Figure 1. This, however, would be premature; the lack of concordance between the expected relationships defined by the DAG and the correlations that test them could be due to one or more of several explanations:

- *The causal diagram could be drawn incorrectly for the outcome*. The first and most obvious reason a DAG is not validated by empirical data is that the DAG is fundamentally incorrect. This could be due to incorrect causal relationships drawn between variables, the lack of needed connections between variables, the erroneous inclusion of irrelevant variables, or the omission of needed explanatory variables or confounders (See Section 4.2 below for further discussion).
- *Analogs could have a different mechanism*. In this scenario the causal diagram is correct for the real-world system it explains, but incorrect for a particular analog dataset that is attempting to approximate it. In other words, the analog is an imperfect simulation of the causal mechanism under study. This is of the utmost concern when using animal models to approximate human exposures (e.g., sending rodents to space), when there may be interspecies differences in physiology, or when humans experience analog exposures (e.g., bed rest studies). In these situations, the correct DAG for the analog could have different nodes and/or slightly different causal mechanisms. For example, in this DAG the terms, bone mass, trabecular microarchitecture, and bone strength were originally modeled as bone density, bone structure, and skeletal fragility in the HSRB official DAG. Where “strength” may be a corollary of fragility, the effects of brittleness as opposed to a load-based conception of strength may impact these results.
- *Variables used in the testing may not be appropriate measures of the random variables on the causal diagram*. In this scenario, the DAG is drawn correctly, any analog in use is a good representation of the real causal system, but one or more measures in the data are inappropriate to represent random variables on the DAG. This can be because the measure is capturing the wrong phenomenon, or because it is measuring the correct phenomenon at the wrong time or in the wrong way.

Among the set of marginal correlations (Figure 2), there were two that were directionally inconsistent, two that lacked sufficient magnitude to be considered non-zero, and two that were lacking in both criteria: directionally inconsistent and of insufficient magnitude. Blending expert knowledge with the details of our results, rather than conclude that the DAG is incorrect, we believe that the measure of bone resorption in the Ko et al. dataset is an inadequate measure (Scenario C from above). We come to this conclusion for several reasons.

First, the correlational failures are highly specific to one dataset and one variable: five out of six failed marginal correlations and five out of six failed conditional independencies were in the Ko dataset and involved bone resorption. Next, three out of the six failed marginal correlations and one of the three failed conditional independencies involving bone resorption in the Ko data were validated by independent tests in the Keune 2016 data. The validation of these relationships in a different setting makes the conceptual relationship more believable and the Ko bone resorption measure more suspect. Finally, the Keune 2016 resorption measure (fluorochrome label length) specifically measures the cumulative extent of bone resorption from the beginning of the study to the end, whereas the measure present in the Ko data is a measure of bone resorption activity, providing a look at this activity at the time of assay only. Essentially, if the rodents in the Ko data had already reached a new homeostasis of bone mass and trabecular microarchitecture by day 28 after exposure, the cross-sectional resorption activity would be comparatively low, and a cumulative resorption measure would be comparatively high. Using the former in place of the latter would mean that the bone resorption measure is not capturing bone resorption in the way the DAG is describing it, and would create the exact opposite of the expected correlations.

### 4.2. Revising Incorrectly Drawn Causal Diagrams

In part (a) of Section 4.1 above, we identified the possibility of having an incorrect visual hypothesis of the variable relationships as a cause of validation failure. It is important to note that failure modes (b) and (c) in Section 4.1 are applicable to the data and its validity in representing variables on the causal diagram (rather than the structure of the diagram itself). These possibilities should be thoroughly investigated and eliminated before entertaining the idea that the diagram may be drawn incorrectly. If the researcher is confident that the causal diagram is incorrect, an attempt may be made to modify it based on the correlation structure of the data used in the validation study. However, this runs the risk of producing a causal diagram completely biased to those data, and repeated validity testing with the same data will only indicate how fully this has been achieved. For these reasons, we advocate that, in the context of HSRB causal diagrams, any modifications should only be proposed when researchers are confident that the data upon which they are basing the modifications are generalizable, such as when they are the result of a systematic literature review, or a large, well-controlled randomized study. Short of this, a more epistemologically sound approach may be to develop the diagram that is most consistent with the currently available data as a proposed update, then perform new studies or otherwise obtain new data to test its validity.

### 4.3. Statistical Precision as a Criterion

As mentioned previously, we have not considered statistical precision to be a validation criterion in this exercise, but included it here for consideration. Had we included it as a criterion, an additional eight marginal correlations in Figure 2 would have failed to be validated on this standard alone, even though the (absolute) magnitudes of all of these correlations were in excess of 0.25. The wide confidence intervals for the estimates derived from the Ko data in Figure 3 would have led to three correlation confidence intervals that were suggestive of non-zero correlation, while the absolute values of the point estimates were all well beyond the 0.15 threshold. These issues illustrate the effect of low statistical power on these intervals, and underscore why we feel that in the context of spaceflight research, statistical precision is a poor criterion.

### 4.4. Limitations of the Method

As with any empirical scientific exercise, the methodology exemplified here has limitations. First among them is that using secondary datasets for DAG validation can be challenging, as few studies will have all the variables needed to validate an entire DAG, and, as in meta-analysis, the variables may take various forms that do not map perfectly to DAG variables. This may require that validations be performed piecemeal, as we have shown here, utilizing datasets to validate only portions of the DAG. This leaves scientists to collect these partial validations and form a judgment from potentially incomplete information. From this perspective, DAG validation—far from being an epistemological silver bullet—merely formalizes our certainties and remaining uncertainties. However, this too has value inasmuch as it defines our understanding and our needs for targeted follow-on mechanistic research. In this case, the identification of knowledge gaps is critical to help scientific program managers consider targeted funding. Additionally, this has significant value in encouraging translational communication between different groups of experts who otherwise may not interact. 

Even if the DAG presented here were to be fully validated by the included rodent data (i.e., all marginal correlations and conditional independencies were met), we would still not definitively declare the DAG to be correct; this is the well-known philosophical “Problem of Induction” revisited [33]. It reminds us that just because there is good correspondence between a causal explanation and the observed data, that does not mean it is the correct explanation, as there are many alternative explanations that could correspond with the data equally well. In the context of causal diagramming, this means there could be many more DAGs whose testable implications could be met by the data. Thus, causal DAGs will ideally be validated with a variety of data over time, building the cumulative evidence for its veracity and inspiring edits and improvements as the data warrant.

### 4.5. DAG Validation and NASA’s Level of Evidence Assessments

DAG validation, such as what we have demonstrated here, is related to the Level of Evidence (LoE) assessment performed by Risk Custodial Teams as part of NASA’s Human System Risk Management Process [5,7]. The LoE process refers to assessing the strength of evidence for each individual arrow on a DAG. It assigns an ordinal rating based on a subset of causal guidelines first defined by epidemiologist Sir A. Bradford Hill in 1965 [34]. Assignment of LoE for an individual causal relationship requires careful review and synthesis of the published literature as well as any unpublished data that NASA has which may be germane. Though this may seem like a highly similar activity to DAG validation, there are important differences. First, LoE assessment is the process of documenting a specific set of heuristic evidentiary criteria for a given causal connection. It attempts to answer the question, “How strong is the evidence that *this single arrow* is correct?” In contrast, the DAG validation process asks, “Does the available evidence support that this *causal system* is correct?” There can be discordance between these two approaches if important causal relationships have been missed in the DAG; all defined arrows may be true, but the DAG may be incorrect for lack of needed causal relationships and/or important variables. Thus, DAG validation should occur after the LoE assessment is at least preliminarily complete, and both assessments should be repeated as often as new evidence warrants. 

However, even if all testable implications were validated, further evidence would still be needed, since all studies used here were of rodents, and none were of humans. Full evaluation of LoE involves an assessment of Quality of Evidence (QoE). While LoE applies to the full set of evidence that informs the justification for a specific edge in a DAG, QoE applies to the specific publications, studies, or datasets used; in this case, it would be the four datasets mentioned above [20,21,22,23]. The 12 guidelines for assessing QoE in the context of animal or cellular studies are provided in Table F-3 in the NASA Human System Risk Management Plan [5]. In short, animal studies are initially considered as “weak” evidence for human system risks based in large part on known translational challenges. Animal studies are eligible for a higher LoE if they demonstrate attention to experimental design and external validation of animal translational research methods, using animal models or cellular or molecular endpoints to generalize from animal to human. Given the pilot nature of this study and the selection of four convenience datasets, a formal evaluation of QoE and LoE has not been performed here. This is because effort was primarily focused on exploring the methods of validation prior to investing resources in a full analysis. However, increasing demonstration of high-quality evidence in the studies/datasets used in future research can help inform assessments of a lack of concordance in these validation approaches. Namely, that studies and datasets that meet high quality guidelines may be more likely to suggest an issue with the DAG itself than with the data being used to validate the DAG. 

While this approach applies specifically to the edge or edges in question within the DAG, the question remains as to whether an LoE rating can apply to a whole DAG or subset of a DAG. This would bring the potential for rapid visualization of knowledge gaps that can be tailored to levels of confidence. In the larger systems domain of risk analysis for spaceflight missions, streamlined communication of locations of high and low confidence in our causal understanding of risk can yield large dividends. Engineers and program managers require a high-level justification for science investments and system trades in human spaceflight. Walking through the nuances of cellular, molecular, or animal research can be counterproductive in trade-space analyses which are already detail-oriented towards systems engineering challenges. The DAG validation processes explored here may eventually provide a pathway that enables better communication of critical information for human system risk contributions to total mission risk.

### 4.6. Future Directions

For cis-Lunar, Mars transit, and Mars surface missions (deep space), the approach the space biomedical field took towards low Earth orbit (LEO) will likely evolve for research and risk analysis/quantification. Indeed, a recent analysis of the proportion of medical/health risk per overall deep space risk mission posture estimated that an initial Mars mission will carry a level of medical health risk that is equal to or greater than the entire mission risk that astronauts faced on the Space Shuttle [11]. Beyond the inclusion of human data (both spaceflight and terrestrial analog) housed in the NASA Life Sciences Data Archive (LSDA), and the curation of more non-human data for DAG validation from sources such as the NASA ALSDA, future spaceflight experiments/grants (whether from NASA or partner agencies) should specifically include experiments that address gaps in knowledge identified by the DAGs, consistent with applied research needs. 

In future studies, the wealth of human spaceflight multiomics data being collected by the NASA Human Research Program (for NASA astronauts), and NASA GeneLab (for animals, humans, and model organisms) must be incorporated into the DAGs to provide molecular-level evidence and relationships. Incorporation of multiomics (e.g., transcriptomics, proteomics, metabolomics) may enable the description of greater detail within and between HSRB nodes. In addition, once scientists generate robust measurement models from existing multiomics data, this will enable the development of new assays and biomarkers that characterize key aspects of risk. Further, machine learning is poised to help analyze multiomics, imaging, and other tabular evidence to add towards this causal DAG risk assessment. Previous work has demonstrated the utility of machine learning for biological causal inference, with the development of CRISP (Causal Relation and Inference Search Platform) for biomedical omics data [35]. Additionally, recent machine learning innovations have led to algorithms which are designed to learn the structure of a DAG from a given set of data, broadening the capabilities for incorporating existing space biological data with the DAG structure [36]. 

In some instances, it may be necessary to omit some detailed variables from multiomics datasets or add some explanatory details to DAGs to understand how the multiomics data and DAG fit together, as the data may speak to causal phenomena that fit “between’’ the existing nodes on the HSRB DAG. Thus, a maximally “open science” community approach is necessary to unite original investigators with external collaborators to facilitate such understanding and ultimately build a robust community consensus on all DAGs. 

The voice and expertise of the communities who produce and reuse these data (and metadata) are critical to standardizing and incorporating space biological and scientific data towards risk probability estimation. Across the 29 DAGs [7] in the effort to validate and enrich them with empirical data, voices must be heard from a wide span of biological and human health researchers. At the core of such a collaborative science campaign is the need to make the necessary biomedical data findable, accessible, interoperable, and reusable for populating DAGs.

## 5. Conclusions

In this example, we have demonstrated a working procedure for the validation of a DAG with data submitted to a NASA data repository (with data not used to originally formulate the DAG). When considering the totality of the evidence these datasets provide, the results largely validate the DAG, and thus affirm the story: skeletal unloading, whether from entering microgravity in space or from partial weightbearing in a terrestrial analog study, stimulates bone resorption and reduces bone formation at some skeletal sites. This in turn leads to lower bone mass and a sparser trabecular microarchitecture, factors which ultimately result in reduced bone strength.

Though this example validated only a small portion of a single larger HSRB risk DAG, the method illustrated here is a general framework for using empirical data to validate causal explanations encoded as a DAG, and as such, may be applied to any DAG for which data can be brought to bear (be that data from NASA data repositories or from other sources).

While the computation of marginal correlations and partial correlations are basic statistical procedures, the interpretation of the results of these tests requires active reasoning and is anything but procedural. Determining why certain implications fail to be validated requires the investigator to wade through the specifics of the results and carefully reason against the possible explanations. It may require multiple iterations of DAG revision and testing, and possibly additional research. This is how NASA’s risk management process is intended to function: existing DAGs prompt evaluation of evidence, which in turn inspires improvements in the causal explanation, which prompts further research and an update to the DAG, and so on, in a virtuous cycle.

Like any other hypothesis, a DAG is never fully proven true, but instead may mount enough evidence in its favor that it becomes no longer worth doubting; we leave it to subject matter experts and other stakeholders to decide when that point has been reached, on a risk-by-risk basis. NASA’s strategy to make long-term human space exploration as safe as possible is to reduce risk through the strategic application of mission and procedure planning, and engineering or medical countermeasures. Whether in complex systems or simple ones, no problem can be effectively solved that is not first effectively understood (and measured). The use of DAGs for modeling complex risk systems and the method we illustrate here for validating them are additional steps in building that understanding.

## Figures and Tables

**Table 1:** Study design characteristics.

Study	Animal Type	Exposure	Control	Bone Assayed	Duration (days)	N
Dubeé [20]	C57BL/6 mice	Spaceflight	Terrestrial	L4 vertebra	37	27
Keune 2015 [22]	Fisher 344 rats	Spaceflight	Terrestrial	Femur	14	24
Keune 2016 [21]	Fisher 344 rats	Spaceflight	Terrestrial	L2 vertebra	14	22
Ko [23]	Wistar rats	Terrestrial: 80% or 60% unloaded	Terrestrial: 0% unloaded	Both femurs	28	33

**Table 2:** Marginal correlations and their expected directions of association.

Variable 1	Variable 2	Expected Direction
Unloading	Bone resorption	Positive
	Bone formation	Negative
	Bone mass	Negative
	Trabecular microarchitecture	Negative
	Bone strength	Negative
Bone resorption	Bone formation	Negative
	Bone mass	Negative
	Trabecular microarchitecture	Negative
	Bone strength	Negative
Bone formation	Bone mass	Positive
	Trabecular microarchitecture	Positive
	Bone strength	Positive
Bone mass	Trabecular microarchitecture	Positive
	Bone strength	Positive
Trabecular microarchitecture	Bone strength	Positive

**Table 3:** Conditional independencies of the DAG in Figure 1.

Variable 1		Variable 2		Conditioning Set
Trabecular microarchitecture	⊥	Unloading	|	Bone formation, Bone resorption
Bone mass	⊥	Unloading	|	Bone formation, Bone resorption
Trabecular microarchitecture	⊥	Bone mass	|	Bone formation, Bone resorption
Bone Strength	⊥	Unloading	|	Bone formation, Bone resorption
Bone Strength	⊥	Unloading	|	Bone mass, Trabecular microarchitecture
Bone Strength	⊥	Bone Formation	|	Bone mass, Trabecular microarchitecture
Bone Strength	⊥	Bone resorption	|	Bone mass, Trabecular microarchitecture
Bone resorption	⊥	Bone formation	|	Skeletal unloading

In the notation used here ⊥ means “is independent of” while | means “given”. Thus, A⊥B|C would be read “A is independent of B, given C”.

## Data Availability

Data used in this work are available through the NASA Ames Life Sciences Data Archive at LSDS-451 (Keune et al., 2015), LSDS-452 (Keune et al., 2016), LSDS-453 (Ko et al., 2020), and LSDS-454 (Alwood, 2016).

## Supplementary Materials

The following supporting information can be downloaded at: https://www.mdpi.com/article/10.3390/biomedicines10092187/s1, Table S1: Composition of component variables.
