# Supplementary material for: Validating Causal Diagrams of Human Health Risks for Spaceflight: An Example Using Bone Data from Rodents
Source: Biomedicines. 2022 Sep 5;10(9):2187. doi: 10.3390/biomedicines10092187 (PMC9496259; doi:10.3390/biomedicines10092187)
Supplement: Supplementary file 1 [file biomedicines-10-02187-s001.zip › biomedicines-1821134-supplementary.pdf]

Component Variable Creation

This appendix describes the methods and results of component variable creation from the 4 source datasets used in this example study. Bracketed references cited here correspond to the references in the main text.

Since there were potentially multiple measures of DAG variables in the 4 datasets, we employed principal component analysis (PCA) as an efficient way to use all available information in a given dataset without redundancy stemming from high levels of correlation among the multiple measures. PCA allows the user to input a set of variables and obtain a set of orthogonal component variables in return. For these calculations we used the function "pca," from the package "psych" in the R Statistical Computing Package[1]**.** This function allowed us to specify how many component variables the function should return, which in turn allowed us to use PCA in a fashion similar to factor analysis: we input all variables that we thought to be germane to a particular concept and obtained a single component variable in return. We initially considered variables for inclusion in each component measure based on the expert opinion of the original research team as to the meaning and purpose of each variable. However, as part of the process of building the components we fit each possible combination of the input variables and selected the one which resulted in the highest proportion of the original variance explained by the component. This served as a heuristic criterion by which to gauge homogeneity of the component, a desirable property given that we sought to define components that represent only one DAG concept at a time. Singular components that explain a high proportion of the variance in the original variables necessarily have high correlation and are therefore likely to be measuring slightly different aspects of the same phenomenon. In contrast, those that require more than a single component to explain a high proportion of the variance are signaling that there is likely information about a different concept in at least one of the variables. The component variables and single-variable measures used to represent nodes on the bone DAG are displayed in Table A1, along with the input variables for the components and the proportion of variance for which the components account.

The data from the study by Dubeé et al. [20] provided measures of bone mass and trabecular microarchitecture taken on lumbar vertebra. We constructed a component variable for bone mass using trabecular thickness and the ratio of bone volume to tissue volume. This component variable was able to account for 90% of the variance in the two original variables. For trabecular microarchitecture we created a component variable that used trabecular number and trabecular separation. The resulting component variable was able to explain 95% of the variance of its inputs.

In the 2016 space flight rat dataset from the study by Keune et al. [21] we were able to create component variables for bone formation and bone resorption, but the ratio of bone volume to tissue volume was the only available measure of bone mass, so no component variable was possible (or necessary). For our measure of bone formation, we extracted a single component out of the cancellous ceased bone formation percentage and the mineral apposition rate. This component was able to explain 77% of the variance in the original two measures. The component for bone resorption used two measures: the post-flight remaining length of a fluorochrome label applied to the bone pre-flight, and the osteoclast perimeter percentage. This component variable captured 78% of the variance in the original variables.

In the 2015 space flight rat dataset from Keune et al. [22], we were able to construct a bone mass component variable and two trabecular microarchitecture component variables. The mass component was derived from two DXA measures: the bone mineral content in milligrams, and the ratio of the bone mineral content to the DXA scanning area. This was able to account for 92% of the original variation in the input variables. Trabecular microarchitecture was represented by two identically constructed component variables, one for the metaphysis of the femur, and one for the epiphysis of the femur. These component variables were created from the site-specific trabecular number and the site-specific trabecular spacing. The composite variable for measurements at the epiphysis was able to explain 97% of the variability of the input variables, while that for the metaphysis explained 94% of the original variance of the input variables. In addition, the dataset had bone volume/tissue volume measures for the epiphysis which, in standardized form, we used as the measure of bone mass.

The variables present in the data from Ko et al. [23] allowed us to make 4 composite variables, one for each of bone mass, bone trabecular changes, bone formation, and bone strength. For bone mass the component was derived from bone volume/tissue volume, bone mineral density, and trabecular number. This component explained 91% of the variance in the original 3 variables. The component variable for bone trabecular microarchitecture was created from trabecular spacing and trabecular number; it was able to account for 97% of the variance of these two inputs. The bone formation component variable was formed from the mineralizing surface to bone surface ratio and the ratio of bone

[1] Revelle, W. *Psych: Procedures for Personality and Psychological Research*; Northwestern University: Evanston, IL, USA, 2022. Available online: https://cran.r-project.org/web/packages/psych/index.html (accessed on 21 June 2022).

formation rate to bone surface area. This component was able to account for 95% of the variance in these input variables. Finally, the composite bone strength variable used the maximum load and the failure load from bone stress testing and was able to explain 94% of the variance in them.

Table S1. Composition of component variables.

	Dubeé et al. 2016		Keune et al. 2015		Keune et al. 2016		Ko et al. 2020	
**DAG Variables**	**Input Variables**	**Variance Explained**	**Input Variables**	**Variance Explained**	**Input Variables**	**Variance Explained**	**Input Variables**	**Variance Explained**
**Bone formation**			• Ceased bone formation %<br>• Mineral apposition rate	77%			• Mineralizing surface/ bone surface<br>• Bone formation rate/ bone surface	95%
**Bone resorption**			• Label length<br>• Osteoclast perimeter %	78%			• Osteoclast surface/ bone surface	N/A
**Bone mass**	• Trabecular thickness<br>• Bone volume/ tissue volume	90%	• Bone volume/ tissue volume	N/A (single variable)	• Bone mineral content (DXA)<br>• Bone mineral density (DXA)	92%	• Bone volume/ tissue volume<br>• Bone mineral density<br>• Trabecular number	91%
**Trabecular microarchitecture**	• Trabecular number<br>• Trabecular separation	95%			• Trabecular number<br>• Trabecular spacing	97%	• Trabecular number<br>• Trabecular spacing	97%
**Bone strength**							• Maximum load<br>• Fail load	94%
